# HIV-2 interaction with cell coreceptors: amino acids within the V1/V2 region of viral envelope are determinant for CCR8, CCR5 and CXCR4 usage

**DOI:** 10.1186/s12977-014-0099-3

**Published:** 2014-11-25

**Authors:** Quirina Santos-Costa, Maria Manuel Lopes, Marta Calado, José Miguel Azevedo-Pereira

**Affiliations:** Host-Pathogen Interaction Unit, Research Institute for Medicines and Pharmaceutical Sciences (iMed.ULisboa), Faculdade de Farmácia, Universidade de Lisboa, Av. Prof. Gama Pinto, 1649-003 Lisboa, Portugal; Instituto de Medicina Molecular (IMM), Faculdade de Medicina, Universidade de Lisboa, Av. Prof. Egas Moniz, 1649-028 Lisboa, Portugal; Centro de Patogénese Molecular, Unidade dos Retrovírus e Infecções Associadas (CPM-URIA), Faculdade de Farmácia, Universidade de Lisboa, Av. Prof. Gama Pinto, 1649-003 Lisboa, Portugal

**Keywords:** HIV-2, Envelope glycoprotein, V1/V2 region, Coreceptor interaction, CCR8, CCR5, CXCR4, Mutagenesis

## Abstract

**Background:**

Human immunodeficiency virus 1 and 2 (HIV-1 and HIV-2) use cellular receptors in distinct ways. Besides a more promiscuous usage of coreceptors by HIV-2 and a more frequent detection of CD4-independent HIV-2 isolates, we have previously identified two HIV-2 isolates (HIV-2_MIC97_ and HIV-2_MJC97_) that do not use the two major HIV coreceptors: CCR5 and CXCR4. All these features suggest that in HIV-2 the Env glycoprotein subunits may have a different structural organization enabling distinct - although probably less efficient - interactions with cellular receptors.

**Results:**

By infectivity assays using GHOST cell line expressing CD4 and CCR8 and blocking experiments using CCR8-specific ligand, I-309, we show that efficient replication of HIV-2_MIC97_ and HIV-2_MJC97_ requires the presence of CCR8 at plasma cell membrane. Additionally, we disclosed the determinants of chemokine receptor usage at the molecular level, and deciphered the amino acids involved in the usage of CCR8 (R8 phenotype) and in the switch from CCR8 to CCR5 or to CCR5/CXCR4 usage (R5 or R5X4 phenotype). The data obtained from site-directed mutagenesis clearly indicates that the main genetic determinants of coreceptor tropism are located within the V1/V2 region of Env surface glycoprotein of these two viruses.

**Conclusions:**

We conclude that a viral population able to use CCR8 and unable to infect CCR5 or CXCR4-positive cells, may exist in some HIV-2 infected individuals during an undefined time period, in the course of the asymptomatic stage of infection. This suggests that *in vivo* alternate molecules might contribute to HIV infection of natural target cells, at least under certain circumstances. Furthermore we provide direct and unequivocal evidence that the usage of CCR8 and the switch from R8 to R5 or R5X4 phenotype is determined by amino acids located in the base and tip of V1 and V2 loops of HIV-2 Env surface glycoprotein.

## Background

Human Immunodeficiency Virus (HIV) envelope (Env) glycoproteins are responsible for initial molecular interactions between HIV and cellular receptors present in plasma membrane. The sequential and specific interaction of Env surface (SU) glycoprotein with CD4 and a member of G-protein coupled receptors (GPCRs), enables the disclosure of a hydrophobic region (called fusion peptide) in Env transmembrane glycoprotein that leads to the fusion of viral envelope with cell membrane [1,2].

The two major GPCRs (known as coreceptors) involved in this complex entry mechanism are CCR5 and CXCR4 [1-6]. However, several other GPCRs have been implicated as coreceptors [7-21], revealing that HIV-1 and HIV-2 isolates can exploit alternate molecules *in vitro* as co-factors for viral entry, raising the possibility that they might contribute to HIV infection of natural target cells *in vivo*. These alternate coreceptors include: CCR2b, CCR3, CCR4, CCR6, CCR8, CCR9, CCR10, CXCR2, CXCR5, CXCR6, CX3CR1, XCR1, FPRL1, GPR1, GPR15, APJ, ChemR23, CXCR7/RDC1, D6, BLTR and US28.

The importance of CCR5 and CXCR4 as HIV coreceptors emanates from (i) the apparent selection of CCR5-using (R5) variants during or soon after HIV-1 mucosal transmission [22]; (ii) the almost exclusive presence of R5 HIV-1 variants during chronic infection; and (iii) the emergence and predominance of CXCR4-using (X4) variants in some patients with advanced HIV-1 disease [23].

We and others have previously demonstrated that *in vitro*, HIV-1 and HIV-2 use cellular receptors in distinct ways, including (i) more promiscuous usage of coreceptors by HIV-2 [24-27]; (ii) more frequent detection of CD4-independent HIV-2 isolates [28-31]; and (iii) identification of CCR5/CXCR4-independent HIV-2 isolates [7,32]. All these features suggest that in HIV-2 the Env glycoprotein subunits may have a different structural organization enabling distinct (although probably less efficient) interactions with cellular receptors.

In HIV-1, the molecular determinants governing coreceptor usage by a certain isolate are located mainly in the third variable region (V3) of SU glycoprotein [33-37]. In HIV-1 subtype B, the presence of basic (positively charged) amino acids at positions 11, 25 and/or 24 (referred to V3 region), an overall charge of V3 region above +6 and the loss of an N-linked glycosylation site within the V3 region are consistently associated with CXCR4 usage [1,2,38-40]. Besides V3 region, also the variable regions 1 and 2 (V1/V2) have been described as cooperating in coreceptor’s choice [1-6,41-43].

In HIV-2, structural and functional studies of envelope glycoproteins regions are much more scarce and in some aspects contradictory. Some studies had claimed an association between V3 loop sequence and CCR5 or CXCR4 usage [7-21,44-47], while others had found no genetic signature underlying coreceptor usage [22,27,48,49]. Particularly, the C-terminal region of the V3 loop, a global net charge above +6 and the presence of mutations in amino acids 18 and 19 (numbers refer to V3 sequence), appear to dictate the ability to use CXCR4 alone or in addition to CCR5 [23,45,47].

During a screening of HIV-2 primary isolates regarding coreceptor usage, we identified two strains obtained from asymptomatic individuals (HIV-2_MIC97_ and HIV-2_MJC97_) that enter target cells independently of CCR5 and CXCR4 coreceptors [7,24-27]. Here the virus-receptors interactions and the SU Env glycoprotein characteristics of these two viruses were further studied in order to (i) decipher which are the molecules used by these isolates to enter target cells; and (ii) which are the molecular determinants underlying the CCR5/CXCR4-independent entry. We provide direct evidence that CCR8 is the cellular receptor engaged as coreceptor by these specific strains. Furthermore, we also demonstrate that the molecular determinants of this phenotype are located in the V1/V2 region of SU Env glycoprotein, providing valuable new insights into the basis of HIV-2 envelope interactions with cellular receptors.

## Results

The interactions between cellular coreceptors and Env glycoproteins from two CCR5/CXCR4-independent HIV-2 strains were investigated. In the first part of this study we identified the CCR8 molecule as the coreceptor used by both strains for viral entry. In the second part, we addressed the determinants of chemokine receptor usage at the molecular level, and deciphered the amino acids involved in the usage of CCR8 and in the switch from CCR8 to CCR5 or to CCR5/CXCR4 usage.

### HIV-2_MIC97_ and HIV-2_MJC97_ uses CCR8 to infect GHOST cell lines and PBMC

Our previous results showed that both HIV-2_MIC97_ and HIV-2_MJC97_ are unable to infect GHOST-CD4 cell lines expressing several coreceptors including CCR5 and CXCR4 [7,28-31]. The CCR5/CXCR4-independent phenotype was demonstrated either in *ccr5* ∆*32/*∆*32* peripheral blood mononuclear cells (PBMC) infection, and by testing the *in vitro* resistance to CCR5 and CXCR4 targeted inhibitors [7,32].

**Figure 1:**
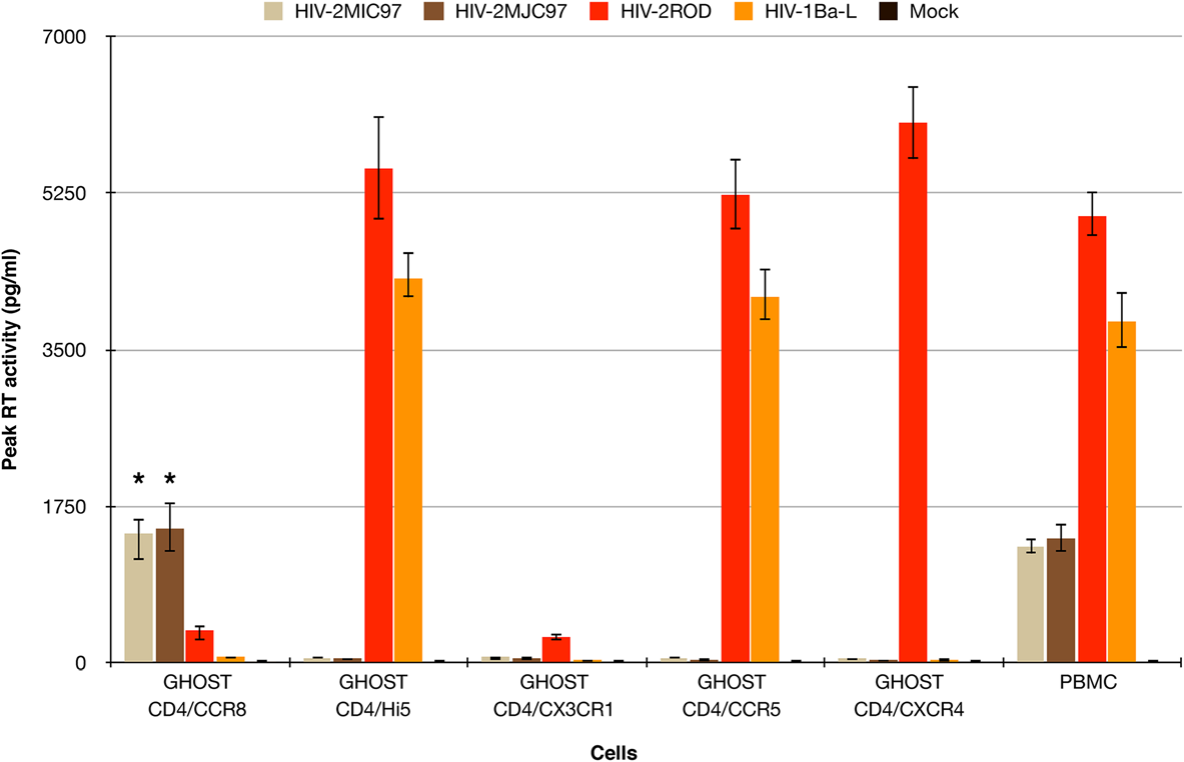
**HIV-2**_**MIC97**_ **and HIV-2**_**MJC97**_ **use CCR8 as coreceptor to infect GHOST-CD4 cells and PBMC.** PBMC and GHOST-CD4 cell lines expressing different coreceptors were exposed to 100 TCID_50_ of each virus; viral replication was quantified by RT activity in culture supernatants during a 12-day period after infection and the highest value of RT activity observed during this time period was used. Results are expressed as the mean of three independent experiments performed in duplicate. Error bars represent the standard error of the mean. A star (*) indicates statistical significant difference (*p* < 0.001) between the means of peak RT activity measured in culture supernatants of GHOST-CD4/CCR8 inoculated with HIV-2_MIC97_ and HIV-2_MJC97_, compared with GHOST-CD4/CCR5, GHOST-CD4/CXCR4, GHOST-CD4/CX3CR1, and GHOST-CD4/Hi5 inoculated with the same viruses.

Since both viruses required the presence of CD4 at cell membrane [7,33-37] together with an unknown coreceptor present in IL-2-activated PBMC, our first goal was to identify this elusive molecule. We initially characterize chemokine receptors usage, by infectivity assays using GHOST-CD4 and U87-CD4 cell lines expressing several chemokine receptors (CCR1, CCR2b, CCR3, CCR5, CXCR4, GPR15 and CXCR6). To further extend these results, we analyzed HIV-2_MIC97_ and HIV-2_MJC97_ utilization of a panel of other potential coreceptors. For this, GHOST-CD4/Hi5, GHOST-CD4/CCR8 and GHOST-CD4/CX3CR1 cell lines were infected with 100 TCID_50_ of each virus. As controls, GHOST-CD4/CCR5 and GHOST-CD4/CXCR4 cell lines and PBMC were included as well as HIV-2_ROD_ (able to use both CXCR4 and CCR5 coreceptors; biotype R5X4) and HIV-1_Ba-L_ (able to use CCR5 coreceptor; biotype R5) viral strains. The results (Figure 1) show that only PBMCs and GHOST-CD4/CCR8 cells are able to support efficiently the replication of HIV-2_MIC97_ and HIV-2_MJC97_ (*p* < 0.001), indicating that these strains require the presence of CCR8 to enter host cells. Viral replication was assessed by measuring RT activity in culture supernatants of infected cells; however, since GHOST cell line carries HIV-2 long terminal repeat (LTR)-driven green fluorescent protein (GFP), we also assessed coreceptor usage by analyzing GFP expression in GHOST-CD4/CCR8, GHOST-CD4/Hi5, GHOST-CD4/CX3CR1, GHOST-CD4/CCR5 and GHOST-CD4/CXCR4 infected cells by fluorescent microscopy (Table 1). This analysis was done in triplicate at days 1, 3, 6, 9 and 12 post-infection and confirms the exclusive usage of CCR8 as coreceptor by HIV-2_MIC97_ and HIV-2_MJC97_.

**Table 1:** **Green fluorescence protein (GFP) expression on GHOST cell lines exposed to different HIV isolates**

**Viruses**	GFP expression in GHOST cells*					
	**GHOST-CD4/CCR5**	**GHOST-CD4/CXCR4**	**GHOST-CD4/CCR8**	**GHOST-CD4/Hi5**	**GHOST-CD4/CX3CR1**	**GHOST-CD4**
**HIV-2MIC97**	-	-	+	-	-	-
**HIV-2MJC97**	-	-	+	-	-	-
**HIV-2ROD**	+	+	-	+	-	-
**HIV-1Ba-L**	+	-	-	+	-	-
**Mock infected**	-	-	-	-	-	-

GHOST cells express either CD4 alone (GHOST-CD4) or CD4 together with different coreceptors.

*LTR-driven GFP expression was analized in GHOST cells by fluorescent microscopy at days 1, 3, 6, 9 and 12 days after infection; + presence of GFP-expressing cells; − absence of GFP-expressing cells.

**Figure 2:**
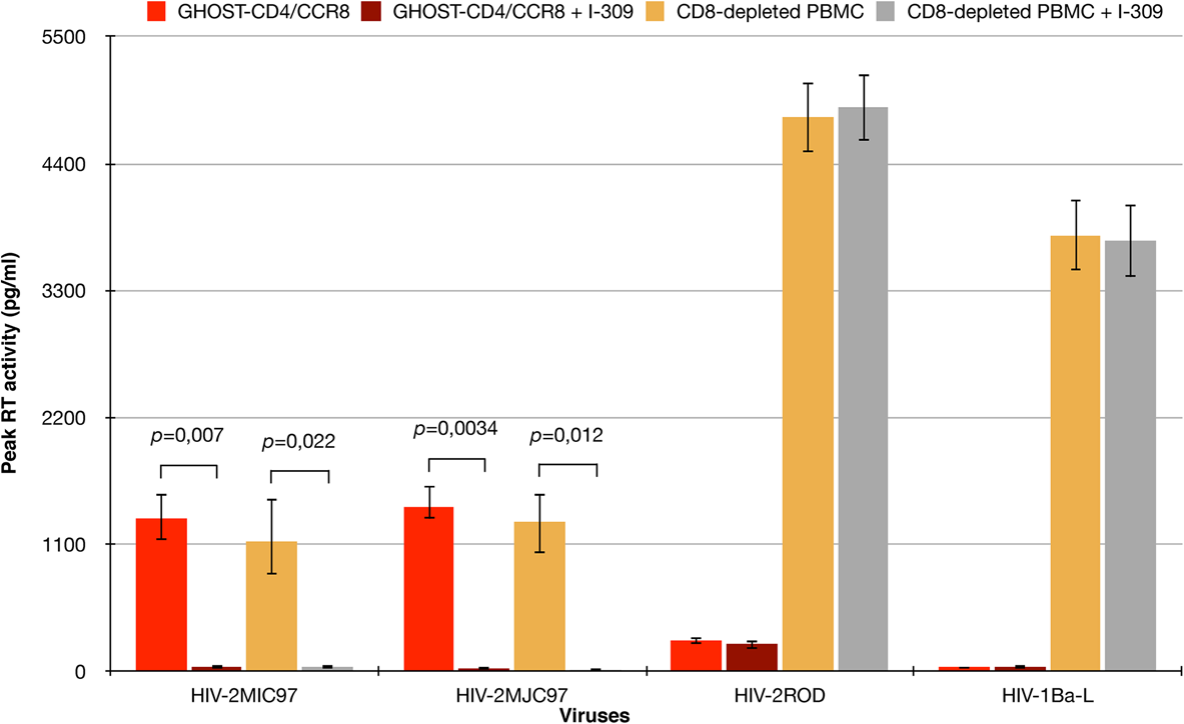
**Specific inhibition of HIV-2**_**MIC97**_ **and HIV-2**_**MJC97**_ **by I-309.** GHOST-CD4/CCR8 cell line and CD8-depleted PBMCs were inoculated with 100 TCID_50_ of HIV-2_MIC97_ and HIV-2_MJC97_ either in the presence or absence of CCR8 ligand, I-309. HIV-2_ROD_ and HIV-1_Ba-L_ were also included as controls. The data are expressed as the mean of three independent experiments performed in duplicate. Error bars represent the standard error of the mean.

The robust usage of CCR8 revealed by GHOST cells assay, prompted us to further confirm the role of this alternate coreceptor in HIV-2_MIC97_ and HIV-2_MJC97_ entry. In order to assure the specificity of CCR8 usage, we incubated 1 × 10^6^ GHOST-CD4/CCR8 cells and PHA-activated CD8-depleted PBMCs with blocking concentrations (100 ng/ml) [50,51] of the CCR8 natural ligand, I-309, prior to the addition of 100 TCID_50_ of each viral strain. As shown in Figure 2, I-309 inhibited the infection of HIV-2_MIC97_ and HIV-2_MJC-97_ replication. The replication of both viruses was significantly reduced (*p* < 0.05) for a concentration of 100 ng/ml in both GHOST-CD4/CCR8 cell line and CD8-depleted PBMCs, further confirming that CCR8 coreceptor was essential for viral entry including in primary cells. As controls, we also tested the ability of I-309 to inhibit the replication of HIV-2_ROD_ and HIV-1_Ba-L_. In both cases the viral replication was not affected by the addition of I-309 (Figure 2).

### Generation of CCR5-using and CCR5/CXCR4-using variants of HIV-2_MIC97_ and HIV-2_MJC97_

The identification of CCR8 as the coreceptor that, together with CD4, enables cell entry by these two strains raised several important questions. One is related to the fact that a population of CCR5-independent variants could maintain a persistent HIV infection *in vivo*. If so, what will be the evolution of this population within the infected patient regarding coreceptor usage? In addition, if this evolution eventually occurs what will be the differences in Env glycoproteins sequences between those isolates? To answer these questions we made efforts to obtain sequential blood samples of the same patients from which we isolated HIV-2_MIC97_ and HIV-2_MJC97_. Unfortunately both patients had left medical outpatient clinic follow-up and therefore it was unfeasible to obtain further samples to help answer these questions.

In order to study the evolution of coreceptor usage (i.e. from CCR8 to CCR5 and/or CXCR4) and thus the HIV-2 envelope glycoproteins determinants that are important in CCR5/CXCR4-independent replication, alternatively we performed an *in vitro* replication adaptation of HIV-2_MIC97_ and HIV-2_MJC97_ to CCR5- or CXCR4-expressing cell lines. The starting viruses for this study was obtained by transfection of 293 T cells with the pROD/MIC-SB and pROD/MJC-SB plasmids [52]. These plasmids contain an infectious HIV-2_ROD_ provirus into which the *env* gene derived from both HIV-2_MIC97_ and HIV-2_MJC97_ isolates, was cloned [52]. The cells used in this experiment were the GHOST-CD4 cell lines individually expressing CCR8, CCR5 or CXCR4. An initial stock of each virus (ROD/MIC-SB and ROD/MJC-SB) was prepared by passing the virus-containing supernatants from transfected 293 T cells in GHOST-CD4/CCR8 cells. Each virus was then used to infect a 90:10 mixture of GHOST-CD4/CCR8:GHOST-CD4/CCR5 and GHOST-CD4/CCR8:GHOST-CD4/CXCR4. At day 12 after infection, culture supernatants were used to infect either a pure population of GHOST-CD4/CCR5 or GHOST-CD4/CXCR4 cells, and a 80:20 mixture of GHOST-CD4/CCR8:GHOST-CD4/CCR5 and GHOST-CD4/CCR8:GHOST-CD4/CXCR4. Virus-containing supernatant from these latter cultures was again used to infect pure GHOST-CD4/CCR5 or GHOST-CD4/CXCR4 cells and a 70:30 mixture of GHOST-CD4/CCR8:GHOST-CD4/CCR5 and GHOST-CD4/CCR8:GHOST-CD4/CXCR4. This procedure was repeated using cell mixtures with increasing proportions of GHOST-CD4/CCR5 or GHOST-CD4/CXCR4 cells, until a ratio 10:90 of GHOST-CD4/CCR8:GHOST-CD4/CCR5 or GHOST-CD4/CCR8:GHOST-CD4/CXCR4 cells. In each step of this adaptation study, the viral supernatants of each inoculated culture (either mixtures or pure populations) were monitored by reverse transcriptase activity in order to detect viral replication. The results reveal that viral progeny was detected in all culture supernatants; however, we could not detect in any occasion the productive infection of pure GHOST-CD4/CCR5 or GHOST-CD4/CXCR4 cells (data not shown). Thus, this serial passage of R8 viruses in a cell population with increasing proportions of CCR5-positive or CXCR4-positive cells did not allowed the *in vitro* selection of mutants with the ability to use either of these coreceptors.

### Construction of V1/V2 mutants by site-directed mutagenesis

Due to inability to generate coreceptor switch mutants *in vitro*, we decided to create and test a panel of isogenic viruses derived from HIV-2_MJC97_ differing only in specific amino acids residues, enabling the analysis of the impact of different Env glycoproteins mutations in coreceptor usage by HIV-2_MJC97_.

**Figure 3:**
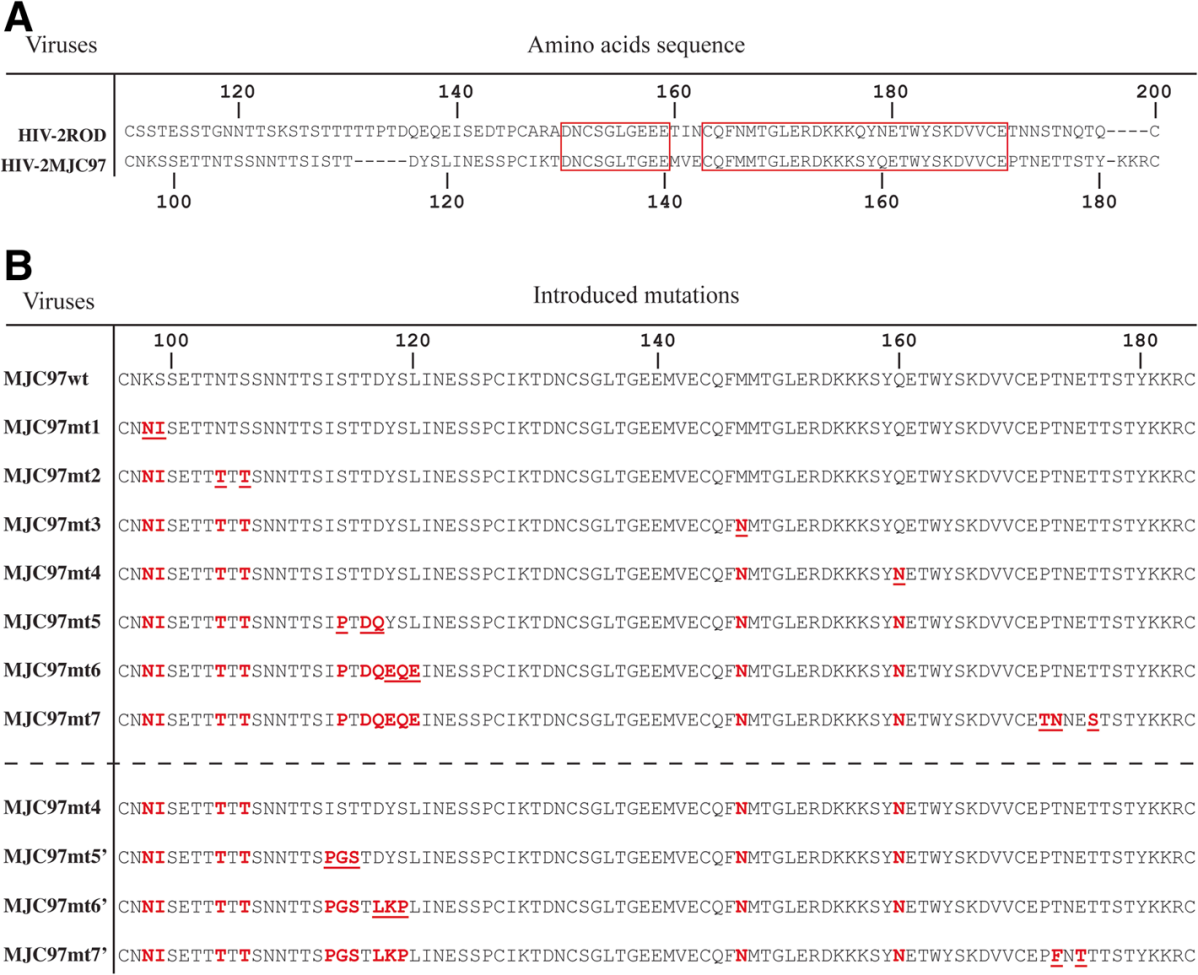
**Amino acid residues changed in V1/V2 region of** ***env*** **glycoprotein by site-directed mutagenesis.** Amino acids are denoted by single-letter code. **(A)** For a better localization of mutated amino acids, the sequence alignment between HIV-2_ROD_ (GenBank accession number: M15390) and HIV-2_MJC97_ (GenBank accession number: EU021092) was included. The red boxes indicate the conserved regions between HIV-2_ROD_ and HIV-2_MJC97_ amino acids sequences. **(B)** The first set of mutants (MJC97mt1 to MJC97mt7) was obtained by sequential mutagenesis starting in the non-mutated recombinant virus, MJC97wt. The second set of mutants (MJC97mt5′ to MJC97mt7′) was derived from the V1/V2 of MJC97mt4. For each sequential mutant, underlined red letters represents the newly changed amino acids residues, while the non-underlined red letters denote mutations that were previously added. Amino acids residues (in panel **A** and **B**) were numbered according to HIV-2_ROD_ (GenBank accession number: M15390) or HIV-2_MJC97_ sequence (GenBank accession number: EU021092).

Previously, we described that *env*-chimeric viruses derived from HIV-2_ROD_ with the SU glycoprotein from either HIV-2_MIC97_ or HIV-2_MJC97_ were unable to infect CD4/CCR5 or CD4/CXCR4 expressing cells, indicating that the C1-C4 region of SU glycoprotein was the only determinant of CCR5/CXCR4-independent phenotype [52]. We also found by comparative *env* gene sequence analysis, that HIV-2_MIC97_ and HIV-2_MJC97_ show remarkable differences in primary amino acid sequence, particularly in the V1/V2 region of each SU glycoproteins [49]. Not surprisingly, but worth noting, despite the differences observed in V1/V2 region we could not identify any discrete sequence signatures that could be hypothetically assigned to the phenotype presented by these two strains [49]. To gain deeper insights into the potential role of V1/V2 domain of Env glycoprotein with regard to coreceptor usage we constructed a variety of different recombinant viruses, all derived from an *env*-chimeric virus (ROD/MJC-SA) described earlier [52] that contains the C1-C4 region of HIV-2_MJC97_*env* gene inserted into the HIV-2_ROD_ backbone by homologous substitution using an infectious molecular clone derived from pROD10 [28]. Multi-site directed mutagenesis of the V1/V2 domain of ROD/MJC-SA *env* was performed targeting the base and the tip of V1 and V2 loops. The details of mutations introduced in each recombinant virus are presented in Table 2 and Figure 3. The first set of mutated viruses (MJC97mt1 to MJC97mt7) was obtained by sequential mutagenesis starting from the V1/V2 *env* region of wild type ROD/MJC-SA (MJC97wt). The second set of mutants (MJC97mt5′ to MJC97mt7′) was derived from the V1/V2 of MJC97mt4. Following each mutagenesis step, the C1-C4 coding region was sequenced to confirm that only the desired changes were introduced.

**Table 2:** **Details of site-directed mutagenesis on selected amino acids in V1/V2 regions of HIV-2**_**MJC97**_ **envelope glycoprotein**

	**Mutations**	
**Mutants identification**	Mutated amino acids*	Amino acids positions**
**MJC97mt1**	N,I	98,99
**MJC97mt2**	T_T	104_106
**MJC97mt3**	N	147
**MJC97mt4**	N	160
**MJC97mt5**	P_D,Q	114_116,117
**MJC97mt6**	E,Q,E	118,119,120
**MJC97mt7**	T,N_ _S	172,173_ _176
**MJC97mt5′**	P,G,S	113,114,115
**MJC97mt6′**	L,K,P	117,118,119
**MJC97mt7′**	F_T	173_175

*Mutated amino acids are referred by single letter code separated by commas (,) if contiguous, or by an underscore (_), if separated by a non-mutated amino acid. See Figure 3 for further details.

**Numbers denote the position of mutated amino acids referred to envelope glycoprotein sequence of HIV-2_MJC97_ (GenBank accession number: EU021092).

**Figure 4:**
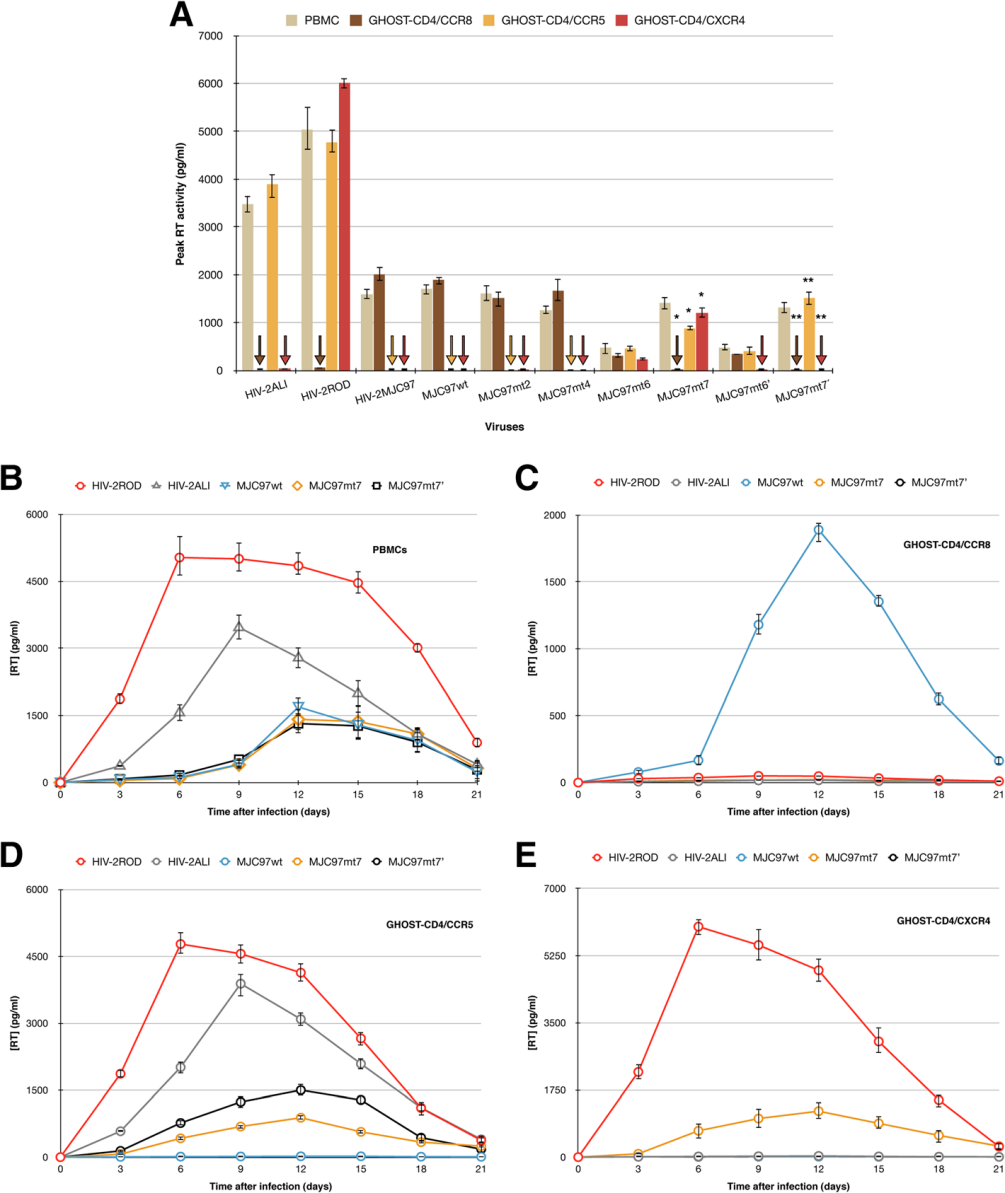
**Effect of sequential mutations in V1/V2 region of HIV-2**_**MJC97**_ ***env*** **glycoprotein on coreceptor usage.** Stocks of each mutated virus (the details of these mutants are described in Figure 3) were used to infect PHA-activated PBMCs and GHOST-CD4 cell lines individually expressing CCR8, CCR5, and CXCR4 coreceptors. **(A)** Viral replication was followed-up for 12 days by assessing RT activity in culture supernatants of infected cells. The highest value of RT activity observed during this time period was used. A star (*) indicates statistical significant difference (*p* < 0.001) between the means of peak RT activity measured in culture supernatants of GHOST-CD4/CCR8, GHOST-CD4/CCR5 and GHOST-CD4/CXCR4 inoculated with MJC97mt7 compared to the same cells inoculated with MJC97wt. Conversely, a double star (**) denotes statistical significant difference (*p* < 0.001) between the means of peak RT activity measured in culture supernatants of GHOST-CD4/CCR8, GHOST-CD4/CCR5 and GHOST-CD4/CXCR4 inoculated with MJC97mt7′ compared to the same cells inoculated with MJC97wt. The strains HIV-2_ALI_ (R5), HIV-2_ROD_ (R5X4) and HIV-2_MJC97_ (R8) were used as controls. Replication kinetics of MJC97wt was compared to mutant viruses that switch from CCR8 usage to CCR5/CXCR4 (MJC97mt7) or to CCR5 (MJC97mt7′); HIV-2ROD and HIV-2ALI strains were also included as controls. The replication kinetics, assessed by RT activity in culture supernatants, was followed up during 21 days and was performed in PHA-activated PBMCs **(B)**, GHOST-CD4/CCR8 **(C)**, GHOST-CD4/CCR5 **(D)** and GHOST-CD4/CXCR4 **(E)**. In all experiments, results are expressed as the mean of three independent experiments performed in duplicate. Error bars represent the standard error of the mean.

The rationale for the selected mutagenesis was based in the *env* sequence analysis and in the discrepancies observed between the V1/V2 coding sequences of HIV-2_MJC97_ (GenBank Accession No. EU021092) and those from R5-tropic HIV-2_ALI_ (GenBank Accession No. AF082339) [28,30] and R5/X4 HIV-2_ROD_ strains (GenBank Accession No. M15390) [53]. Using this approach we were able to construct a total of 10 different recombinant coding sequences (Figure 3) containing combined mutations in the V1/V2 region, all included in the genetic backbone of the R5/X4-tropic HIV-2_ROD_ strain [52]. The mutated recombinant coding sequences were used to reconstitute replication-competent viruses by transfection in 293 T cells, and further expanded in IL2-stimulated PBMC. Although all chimeric viruses were able to replicate in PBMC, the replication efficiency was importantly reduced in some mutated viruses, namely MJC97mt6 and MJC97mt6′ (Figure 4), indicating that the modification of certain V1/V2 motifs indeed strongly affect the replication fitness of recombinant viruses.

### Coreceptor usage by mutated recombinant viruses

To gain greater definition into the nature of the relationship between V1/V2 and cellular receptors engagement in HIV-2, an initial stock of mutated viruses (MJC97mt2, MJC97mt4, MJC97mt6, MJC97mt7, MJC97mt6′ and MJC97mt7′) was prepared by passing each viral-containing supernatants from transfected 293 T cells in IL2-stimulated PBMC. Each replication-competent virus stocks were used to analyze coreceptor usage patterns on GHOST-CD4 cells expressing different coreceptors (CCR5, CXCR4 and CCR8). The objective was to assess the potential implications of the sequential mutations introduced in the V1/V2 regions on coreceptor choice. Viral stocks from MJC97mt2, MJC97mt4, MJC97mt6, MJC97mt7, MJC97mt6′ and MJC97mt7′ were inoculated in GHOST cells and PBMC, and viral replication was followed-up for 12 days by measuring RT activity in culture supernatants of infected cells. The mean of peak RT activity of three independent experiments performed in duplicate was calculated. As controls, GHOST cells and PBMC were also inoculated with HIV-2_MIC97_, HIV-2_MJC97_, R5 strain HIV-2_ALI_ and the R5/X4 strain HIV-2_ROD_, obtained after transfection of 293 T cells with pROD10, an infectious molecular clone of HIV-2_ROD_ [54]. As shown in Figure 4 (panel A), MJC97mt7 clearly show a switch in coreceptor usage from CCR8 to CCR5/CXCR4 (*p* < 0.001), while MJC97mt7′ changed from CCR8 to CCR5 usage (*p* < 0.001). Noteworthy, all the other mutants maintained their ability to use CCR8, similarly to the wild-type (MJC97wt), although some of them noticeable with less efficiency (e.g. MJC97mt6 and MJC97mt6′).

To further assess the viral replication efficiency of MJC97mt7 and MJC97mt7′ we infected PBMCs and GHOST-CD4 cell lines individually expressing CCR8, CCR5 and CXCR4. Besides the mutants that effectively changed from R8 to R5X4 (MJC97mt7) and from R8 to R5 (MJC97mt7′), we also included MJC97wt, HIV-2_ROD_ and HIV-2_ALI_ (as controls). The results summarized in Figure 4 (panels B to E), indicates that the coreceptor switch from CCR8 (MJC97wt) to CCR5 (MJC97mt7′) or to CCR5/CXCR4 (MJC97mt7) was not followed by an increase in replication kinetics, regardless the mutated virus considered, suggesting that different regions besides V1/V2 influence replication kinetics. This is in accordance with our previous observation pointing to the transmembrane domain of Env glycoproteins as major determinant for the lower replication rate observed in both HIV-2_MIC97_ and HIV-2_MJC97_ [52]. Additionally, we also notice that the levels of RT activity in GHOST cell lines and PBMCs were not significantly different. Considering the described higher cellular densities of CD4 and coreceptor molecules in GHOST cells [55,56] and since the concentration of receptors on cell surface has a direct impact in viral entry events [57] it was surprising this similarity in viral replication. However, we do not access viral entry efficiency but instead we used *de novo* viral production as marker of efficient infection. The production of viral particles *de novo* is the result of many other factors besides viral entry through the interaction with cell receptors. Accordingly, replication efficiency is the result of the overall contribution of several events besides viral entry step. A possible explanation for the similar levels of RT activity in GHOST cell lines and PBMCs is that the minor cell receptors expression in PBMC is compensated by more efficient intracellular events during the entire replication cycle compared to GHOST cell lines.

**Figure 5:**
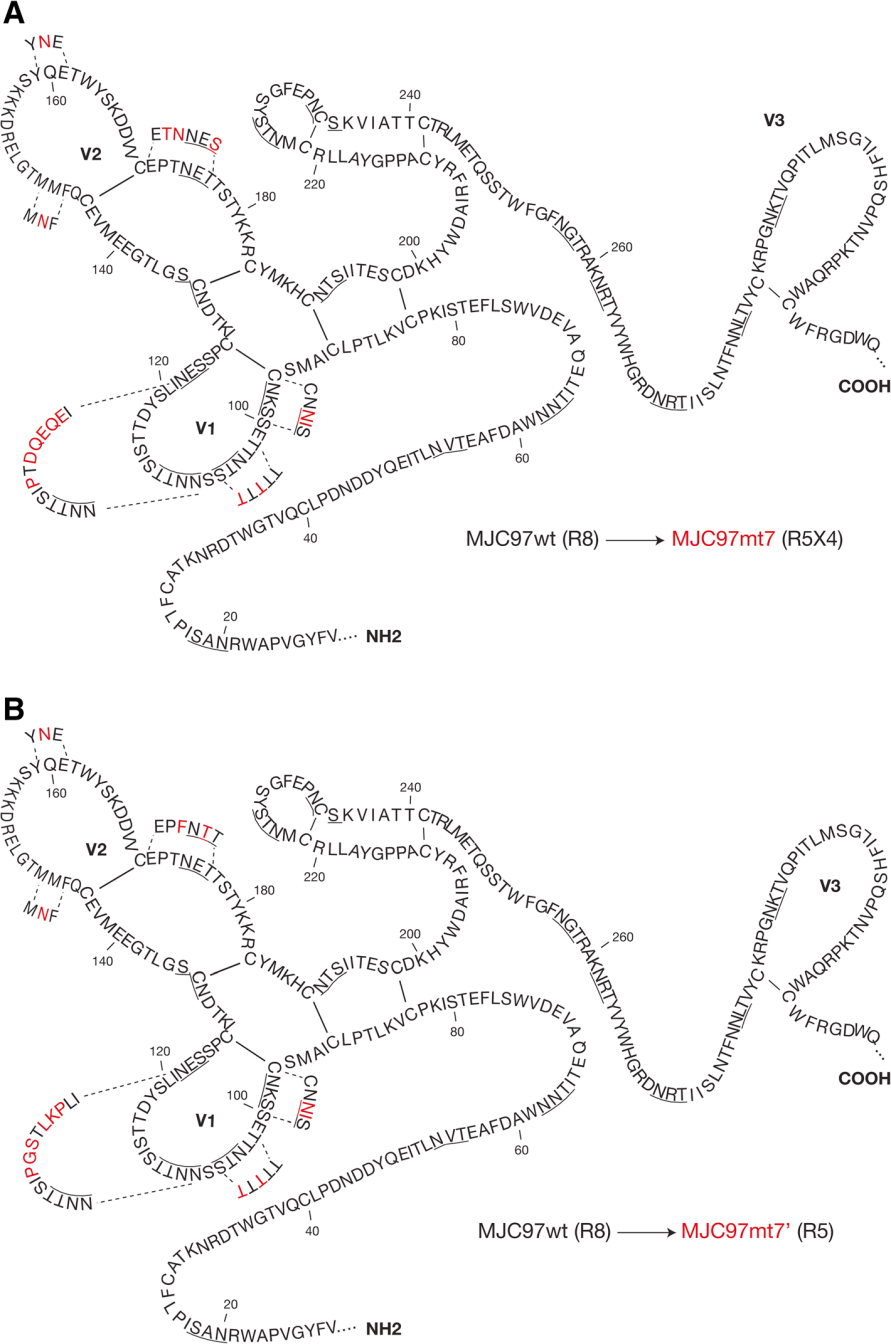
**Location of amino acids residues involved in coreceptor usage.** Schematic representation of the envelope SU glycoprotein of HIV-2_MJC97_ putative secondary structure spanning from C1 to V3 regions. The amino acid sequence of HIV-2_MJC97_ (MJC97wt; R8) are represented in black; the mutated amino acids present in MJC97mt7 (R5X4; panel **A**) and MJC97mt7′ (R5; panel **B**) are represented in red. Amino acids are denoted by single-letter code. The underlined amino acids represent potential glycosylation sites linked to asparagine (N) as defined using the LANL N-glycosite program (http://www.hiv.lanl.gov/content/sequence/GLYCOSITE/glycosite.html). Amino acids residues were numbered according to HIV-2_MJC97_ sequence (GenBank accession number: EU021092). This schematic representation was based on previous data regarding epitope and cysteine loops mapping [58,59].

Based on previous reports addressing antibody binding and cysteine loops mapping of HIV-2 SU glycoprotein [58,59], we located the mutations of MJC97mt7 and MJC97mt7′ either on the tip or base of V1 and V2 loops (Figure 5).

Interestingly, although MJC97mt6 and MJC97mt6′ still maintained the ability to infect GHOST-CD4/CCR8 cells, they also show the ability to infect GHOST-CD4/CCR5 (MJC97mt6′) or GHOST-CD4/CCR5 and GHOST-CD4/CXCR4 cells (MJC97mt6). This transitional state from R8 to R5X4 or R8 to R5 phenotype was acquired after mutational change of the tip of V1 region (Figures 3 and 5). Noteworthy, both MJC97mt6 and MJC97mt6′ show a decreased replication in GHOST-CD4/CCR8 compared to MJC97wt (*p* < 0.001 in both cases).

**Figure 6:**
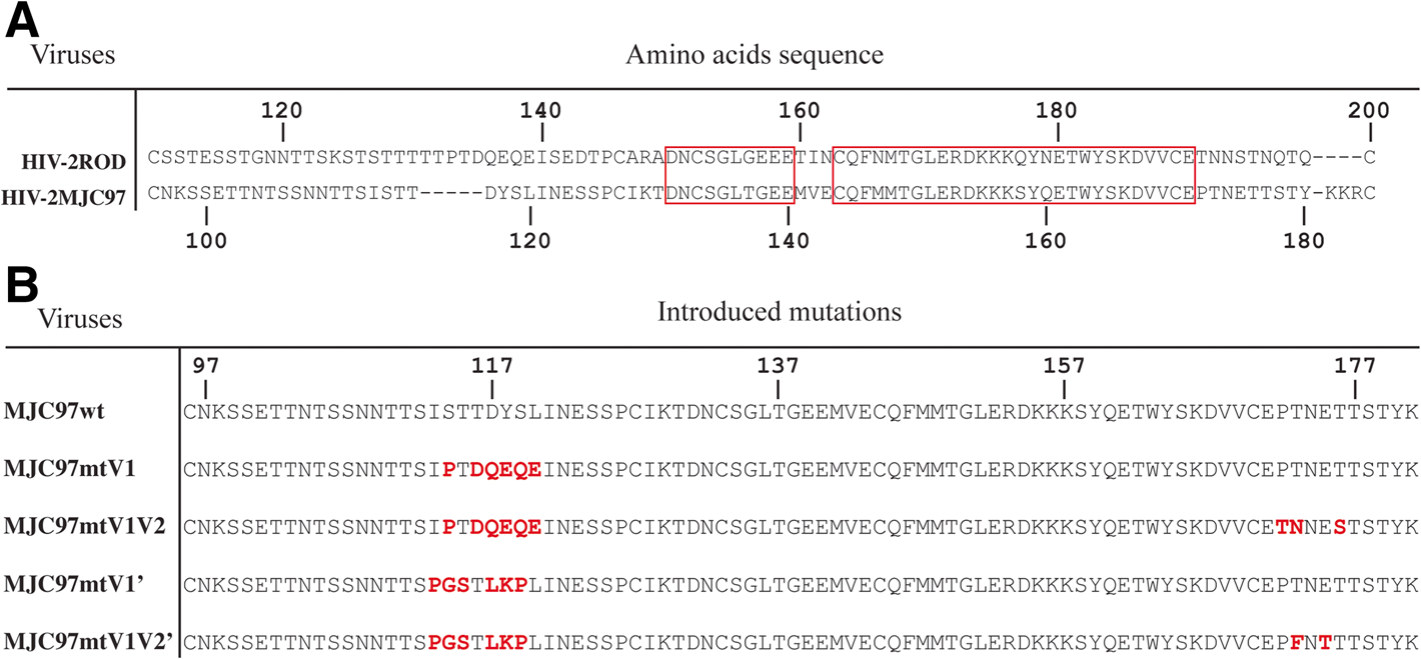
**Amino acid sequences of mutants targeting the tip of V1 region and the base of V2 loop. (A)** For a better localization of mutated amino acids, the sequence alignment between HIV-2_ROD_ (GenBank accession number: M15390) and HIV-2_MJC97_ (GenBank accession number: EU021092) was included. The red boxes indicate the conserved regions between HIV-2_ROD_ and HIV-2_MJC97_ amino acids sequences. **(B)** The tip of V1 of MJC97wt has the sequon ISTTDYSL (amino acids residues 113–120) that was changed to I*P*T*DQEQE* (MJC97mtV1) or *PGS*T*LKP*L (MJC97mtV1′). The MJC97mtV1V2 was obtained by replacing the sequon PTNET (amino acids residues 172–176) in the base of V2 loop of MJC97mtV1 by *TN*NE*S*; MJC97V1V2′ was originated by changing the referred sequon of MJC97mtV1′ by P*F*N*T*T. Amino acids residues (in panel **A** and **B**) were numbered according to HIV-2_ROD_ (GenBank accession number: M15390) or HIV-2_MJC97_ sequence (GenBank accession number: EU021092).

**Figure 7:**
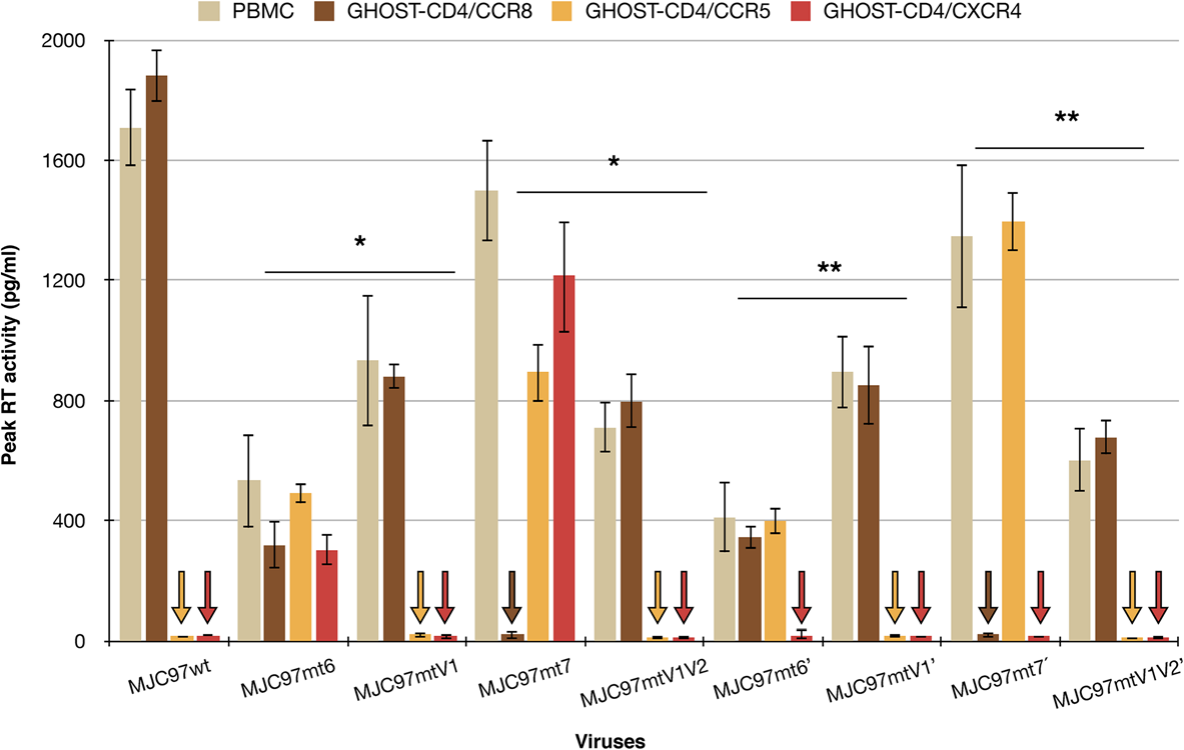
**Coreceptor usage of mutants targeting the tip of V1 region and the base of V2 loop.** Recombinant viruses with mutations targeting the tip of V1 region (MJC97mtV1 and MJC97mtV1′) or with additional mutations in the base of V2 loop (MJC97mtV1V2 and MJC97mtV1V2′). The details of these two sets of mutants are described in Figure 6. Viral replication was followed-up for 12 days by assessing RT activity in culture supernatants of infected cells. The highest value of RT activity observed during this time period was used. Results are expressed as the mean of three independent experiments performed in duplicate. Error bars represent the standard error of the mean. A star (*) indicates statistical significant difference (*p* < 0.001) between the means of peak RT activity measured in culture supernatants of GHOST-CD4/CCR8, GHOST-CD4/CCR5 and GHOST-CD4/CXCR4 inoculated with MJC97mt6 and MJC97mtV1 or MJC97mt7 and MJC97mtV1V2. A double star (**) indicates statistical significant difference (*p* < 0.001) between the means of peak RT activity measured in culture supernatants of GHOST-CD4/CCR8 and GHOST-CD4/CCR5 inoculated with MJC97mt6′ and MJC97mtV1′ or MJC97mt7′ and MJC97mtV1V2′.

These results suggest that amino acid residues in the crown of V1 loop are a critical determinant for the switch from CCR8 to CCR5 or CCR5/CXCR4 usage and thus for Env-coreceptor interactions. The mutated amino acids encompass the motif ISTTDYSL (amino acids residues 113 to 120 according to HIV-2_MJC97_ sequence, GenBank Accession No. EU021092; Figures 3 and 5) present in MJC97wt (R8 phenotype) that was changed to *PGS*T*LKP*L (the mutated amino acids correspond to the italicized letters) present in MJC97mt6′ (R8R5 phenotype) and MJC97mt7′ (R5 phenotype) or to I*P*T*DQEQE* present in MJC97mt6 (R8R5X4) and MJC97mt7 (R5X4 phenotype). To further address the suggested critical role of the V1 crown as molecular determinant for viral coreceptor-tropism switch, we constructed four additional mutants (Figure 6). In two of these mutants only the motif ISTTDYSL was changed: MJC97mtV1 carrying the sequon I*P*T*DQEQE*; and MJC97mtV1′ carrying the sequon *PGS*T*LKP*L (the mutated amino acids correspond to the italicized letters). In the other two mutants we combined the referred mutations in the tip of V1 region with additional mutations located at the base of the V2 region, where the sequon PTNET (MJC97wt) was replaced in MJC97mtV1 by *TN*NE*S* (originating MJC97mtV1V2); and in MJC97mtV1′ was replaced by P*F*N*T*T (originating MJC97mtV1V2′). As shown in Figure 7 mutating the tip of V1 region alone or combined with mutations at the base of V2 region, did not confer the ability to efficiently use CCR5 (MJC97mtV1′ or MJC97mtV1V2′) or CCR5 and CXCR4 (MJC97mtV1 or MJC97mtV1V2). Together these results clearly indicate that although coreceptor switch is dependent on mutations in ISTTDYSL sequon it requires additional changes in other regions of V1/V2. Conversely, they also emphasize that changes in a single amino acid - even if it is relevant - can have phenotypic consequences that are context dependent, relying on the simultaneous presence of additional mutation that may be required to stabilize the interaction with a given coreceptor. The need for cooperating mutations and the viral fitness disadvantage of intermediate mutants - as shown in MJC97mt6 and MJC97mt6′ - when compared with the initial viruses (*p* < 0.001), could help explain the unsuccessful *in vitro* adaptation experiments.

In conclusion, our data clearly show that the main genetic determinants of coreceptor tropism are located within the V1/V2 region of SU glycoprotein and include the crown of V1 loop and discrete amino acids present in: (i) the tip of V2; (ii) the base of V1; and (iii) the base of V2. This emphasizes the plasticity with which SU glycoproteins can interact with coreceptors and the variety of molecular determinants that can influence this interaction.

## Discussion

HIV entry into susceptible cells requires the presence of CD4 and a chemokine receptor (coreceptor), usually CCR5 or CXCR4. However, other alternate coreceptors have been described and may play an effective role in HIV-1 and HIV-2 entry.

We previously showed that two HIV-2 primary isolates could infect susceptible cells by a CCR5/CXCR4-independent pathway [7]. Herein, we extend the study of this model aiming the disclosure of: (i) the alternate coreceptor used by these HIV-2 isolates (HIV-2_MIC97_ and HIV-2_MJC97_) and (ii) the amino acids residues responsible for the CCR5/CXCR4-independent entry.

In the first part of the study we identified CCR8 as the coreceptor used by HIV-2_MIC97_ and HIV-2_MJC97_ to infect host cells. The restrict use of CCR8 by HIV-2_MIC97_ and HIV-2_MJC97_ indicates that a viral population present in HIV-2 infected individuals during asymptomatic stage could use other coreceptors besides or instead CCR5 and CXCR4. Although these two chemokine receptors are considered as the major coreceptors for HIV entry into host cells, the possibility that alternative molecules could have physiological relevance *in vivo* as cofactors for HIV infection remains open. In fact, a growing body of evidence indicates that both HIV-1 and HIV-2 isolates can use distinct coreceptors *in vitr*o together with or alternatively to CCR5 and CXCR4 [7-11,14,16,17,19,20,32,60,61]. In particular, CCR8 usage was referred in earlier reports either in indicator cell lines (e.g. GHOST, U87 or NP2 cells) or in primary lymphocytes [9,14,17,19,29,51,62-66]. More recently, we studied the relevance of CCR8 as an effective coreceptor for HIV-1 and HIV-2 primary isolates [8] and interestingly we found that CCR8 could be frequently used (in addition to CCR5, CXCR4, or both), by HIV-1 and HIV-2 primary isolates. Noteworthy, the cellular and tissue distribution of CCR8 includes cells that are major targets for HIV infection, e.g. monocytes, thymocytes and CD4+ memory T-cells [67-71]. Thus, CCR8 usage does not necessarily implies a change in HIV cell tropism compared to CCR5 or CXCR4 usage. As a result of this expression pattern, and based on the significant proportion of HIV strains able to use CCR8 to enter target cells [8,9,14,17,51], we may considerer it as a potential alternative HIV coreceptor *in vivo* contributing to infection of natural target cells, at least under certain circumstances. This may be even more likely in HIV-2 since in this model the usage of cell receptors seems to be much more complex, as suggested by the identification of HIV-2 strains characterized by: (i) a CCR5/CXCR4-independent entry; (ii) a broader coreceptor usage compared to HIV-1; and (iii) a CD4-independent infection of host cells (reviewed in [72-74]).

The restricted use of CCR8 by HIV-2_MIC97_ and HIV-2_MJC97_ is an apparent paradox based on the general assumption that HIV-2 isolates have a broad profile of coreceptor usage [24-26,75]. However, as a consequence of technical hindrance concerning *in vitro* HIV-2 isolation from asymptomatic aviremic patients, the majority of HIV-2 data regarding coreceptors usage has been derived from viruses obtained from patients in advanced disease stages, where more pathogenic variants with broader coreceptor usage could be present, leading to a bias in the viral population that was preferentially isolated. In contrast, HIV-2_MIC97_ and HIV-2_MJC97_ were isolated from two asymptomatic patients with undetectable viremia and normal T-CD4+ cell counts (1078 and 896 cells/mm^3^, respectively). Interestingly, another example of a CCR5/CXCR4-independent HIV-2 isolate was also obtained from an asymptomatic individual [32]. As referred, HIV-2 and HIV-1 infections are strikingly different during this period. At this early stage, HIV-2 infection resembles a natural long-term non-progressive infection as observed in those rare HIV-1 “elite controllers” [76,77]. The reasons for this milder and less virulent infection are multi-factorial encompassing distinct mechanisms triggered by virus-host interactions, namely during cellular receptor’s engagement.

The data presented here reveal that in humans a persistent lentiviral infection could be maintained by variants that do not use CCR5 or CXCR4 coreceptors. Similar observations have been reported in simian immunodeficiency virus (SIV) model, where some isolates have been described that do not use CCR5 to infect simian primary lymphocytes [78,79]; instead, these isolates use alternative coreceptors such as CXCR6, GPR15 and CCR2b [78,80,81]. Coreceptors usage other than CCR5 and CXCR4 has been considered of limited importance for HIV infection *in vitro* and *in vivo*. Particularly, the use of CCR5 coreceptor seems to be a hallmark in HIV-1 pathogenesis and in human transmission (reviewed in [82]). The predominance of R5 strains throughout the asymptomatic stage and in some patients with more advanced disease, suggest that these variants may be more adapted to escape immune surveillance mechanisms or that they could infect long-lived cell reservoirs, providing long-lasting R5 viruses production. Additionally, it has been suggested that soon after sexual transmission only R5 viruses (or occasionally dual tropic viruses, R5X4) are transmitted, regardless the overall composition of initial inoculum (reviewed in [22,83]). However, a recent observation revealed that a transmitted/founder HIV-1 was unable to use either CCR5 or CXCR4 to infect CD4+ cell lines and peripheral blood mononuclear cells [13]. Instead, alternate coreceptors (i.e. GPR15, APJ and FPRL-1) were efficiently used, emphasizing the notion that “rare” or “minor” coreceptors could be used *in vivo* in some circumstances or in some cell types, including at the time or soon after transmission to a new host. In HIV-2 no data exists regarding transmitted/founder viruses or the characteristics of viral dynamics during acute infection. It is conceivable that the same mechanisms proposed for HIV-1 could also be relevant in HIV-2 transmission. Unfortunately we could not obtain data regarding route and date of transmission nor sequential blood samples of the patients from which we isolated HIV-2_MIC97_ and HIV-2_MJC97_ in order to ascertain what would be the evolution of this viral population *in vivo*. Nevertheless, our present data, together with previous reports [7,32] raise the possibility that, *in vivo*, CCR5 usage ability, required for an efficient *in vivo* infection, could be acquired, from an initial population of CCR5/CXCR4-independent viruses, in addition or in alternative to the initial receptors used.

In the second part of this study we mapped HIV-2 envelope glycoproteins determinants of CCR8 coreceptor usage, and the amino acids residues involved in coreceptor switch from R8 to R5 or R8 to R5X4. Our data provided the basis for some important conclusions, namely that: (i) the V1/V2 region contains the molecular determinants of coreceptor usage (e.g. CCR8, CCR5 and CCR5/CXCR4); (ii) several mutations are needed to convert a R8 isolate into a R5 or R5X4 variant; (iii) the replication kinetics is not affected by the mutations introduced in V1/V2 region.

In HIV-1, the V3 region of the envelope SU glycoprotein has been directly implicated as the major molecular determinant of coreceptor usage [33-37]. One of the major sequence signatures related to CXCR4 usage (alone or in addition to CCR5) seems to be a higher positive net charge of the V3 region. According to this “rule” a net charge equal to or higher than +6 is associated with CXCR4 usage [42,84-86]. The ability to use the CXCR4 is also related with loss of a putative N-linked glycosylation (PNG) site within the V3 region [40]. Additionally to V3 region, structural studies of SU bound to cellular receptors (CD4 and chemokine receptor) revealed that V1/V2 region of SU glycoprotein is also involved in coreceptor binding, by directly cooperate with the V3 region [40-43,87].

In the HIV-2 model, some studies had claimed an association between different coreceptor usage and specific sequence motifs within V3 region [44-47,88]. All the proposed sequence motifs acting as determinants of coreceptor usage are located in the C-terminal half of the V3 region (aa-18 and aa-36 of V3 loop sequence) and apparently, a global V3 net charge above +6 and the substitution of valine or isoleucine at position 19 are associated with CXCR4 usage alone or in addition to CCR5 [45,47]. However, other reports have failed to intersect the V3 amino acid sequence with coreceptor engagement, suggesting that no singular genetic signature could be proposed to explain different coreceptor usage [27,48,49].

Our data is the first to disclose the role of V1/V2 region in coreceptor engagement during initial HIV-2 interaction with host cell. In fact, using a panel of isogenic mutant viruses we demonstrate that the switch from R8 to R5 or R8 to R5X4 phenotype is determined by amino acids located in the base and tip of V1 and V2 loops. Interestingly, two of the mutations introduced two PNG sites both in the V2 loop. These two additional PNG sites are present in both R5 and R5X4 HIV-2_MJC97_ mutants but absent in the original R8 non-mutated virus. However, these two additional PNG sites did not alter the coreceptor usage (see MJC97mt4 in Figure 4A). There is scant information about the contribution of N-linked glycosylation in HIV-2 tropism and infectivity. However, as observed in HIV-1 [89], the influence of discrete PNG sites is probably context dependent and the same mutations could have different effects in tropism, depending on the overall Env structure and the molecular mechanism modulating binding to cellular receptors.

The way V1/V2 interacts with coreceptors, as well as the spatial organization of different Env structures and the conformational changes that they must undergo during receptor/coreceptor binding, are essentially unknown in HIV-2. Thus, any suggestions withdrawn from our results lack the direct supportive data already available for HIV-1 regarding Env glycoproteins structure in the trimeric native form [90,91]. From these and other previous reports [92-97], several conclusions were made possible, the most important being that in HIV-1 the V1/V2 region, although not essential for viral entry is crucial to escape antibody-mediated neutralization [43,98-103]. This protective role of V1/V2 region derives from the remarkable antigenic variability observed in this region, the presence of several PNG sites and the length variation of V1/V2 region. Due to structural interactions and rearrangements within the HIV-1 oligomeric Env glycoprotein, V1/V2 is also known to play a major role in conformational masking, creating a shield that protects other neutralization-sensitive domains either in the same SU glycoprotein, or in an adjacent SU subunit in the context of the trimeric Env spike complex [99-102].

In HIV-2, uncertainty prevails on which structural interactions and conformational dynamics must exist between different domains of trimeric Env glycoproteins. In addition, HIV-2 Env glycoproteins interactions with cell receptors seems to be much more complex and apparently less clear-cut than in HIV-1 (reviewed in [72-74]), and as supported by the present study, V1/V2 region could also directly and exclusively determine the coreceptor usage. To what extend the mechanisms described for HIV-1 are also dictating the tertiary and quaternary structure of HIV-2 envelope glycoproteins is not understood and neither are the precise contribution of V1/V2 and V3 regions in antibody-mediated neutralization *in vivo* [104-110]. However and worth noting, the V1/V2 region of HIV-2 has long been described as a target for neutralizing MAbs *in vitro*, and the influence of the overall conformation of this region (namely the amino acid composition at the base of the V2 loop) may affect the sensitivity to neutralization [59]; if we assume that this region also elicits host-neutralizing antibodies *in vivo* (as the RV144 vaccine trials against HIV-1 suggested [111]), and is simultaneously determinant for coreceptor engagement, this could constitute a major hindrance to HIV-2 effective replication and may help explain the low viremia and the higher and broader neutralizing capacity observed in sera from HIV-2 infected individuals [104,105,107]. Further studies using HIV-2 isolates obtained from asymptomatic individuals may provide further insights into factors associated with slow disease progression observed in HIV-2 infection.

## Conclusions

In this article we clearly identify CCR8 as the exclusive coreceptor used by two primary isolates obtained from asymptomatic HIV-2 patients, instead of the widely referred CCR5 and CXCR4. In addition, we delved into the molecular interactions between surface envelope glycoprotein and this coreceptor and disclosed the amino acids residues that dictate the CCR8 usage. By site-directed mutagenesis we found that residues in the tip and base of V1/V2 region of surface glycoprotein are both necessary and sufficient to switch from CCR8 to CCR5 or to CCR5/CXCR4 usage.

Our study adds important new clues to the way HIV-2 envelope interacts with host-cells, and provides new insights into the molecular and structural dynamics underlying HIV-2 interaction with host cell coreceptors with direct implications in HIV-2 pathogenesis.

## Methods

### Cells and viruses

Peripheral blood mononuclear cells (PBMCs), from HIV-uninfected donors, homozygous for wild-type *ccr5* gene, were isolated, phytohemaglutinin (PHA)-stimulated and cultured as described [8]. PBMCs used in all experiments reported here were obtained from one single pool of different buffy-coats to avoid inter-individual variations in HIV infection susceptibility. CD8-depleted PBMCs were obtained from PBMCs after removal of CD8+ cells, using magnetic beads coated with anti-CD8 antibody as described [8].

Human osteosarcoma cell lines GHOST expressing CD4 and different coreceptors (GHOST-CD4/Hi5, GHOST-CD4/CCR8, GHOST-CD4/CX3CR1, GHOST-CD4/CCR5 and GHOST-CD4/CXCR4) were obtained through the National Institute of Health (NIH) AIDS Research and Reference Reagent Program, Division of AIDS, NIAID, NIH. These GHOST cell lines were maintained as described earlier [8].

HIV-2_MIC97_ and HIV-2_MJC97_ primary isolates were obtained from PBMCs of infected patients by co-cultivation with PHA-stimulated PBMC. The isolation and initial characterization of HIV-2_MIC97_ and HIV-2_MJC97_ was previously reported [7,49,52]. Primary HIV-2_ALI_ isolate [28,30] was obtained from an early symptomatic patient (stage B2 according to CDC classification system for HIV infection). Two well-characterized laboratory strains, HIV-2_ROD_ [112] and HIV-1_Ba-L_ [113], both isolated from AIDS patients, were used in some experiments as controls. HIV-1_Ba-L_ was obtained through the NIH AIDS Research and Reference Reagent Program, Division of AIDS, NIAID, NIH. Primary HIV-2 viruses were only short-passaged in PHA-stimulated PBMCs cultured in RPMI medium as described [7]. The 50% tissue culture infectious dose (TCID_50_) was determined by standard end-point dilution method (serial 10-fold dilutions in quadruplicate), using PBMC as target cells. Viral replication was monitored in culture supernatants by reverse transcriptase (RT) activity using an enzyme-linked immunosorbent assay (Lenti-RT kit, Cavidi).

### Infectivity assays

Infectivity assays in PBMCs and GHOST cell lines were performed as described [7]. Briefly, cells were seeded into 24-well plates on the day prior to infection, at 1.5 × 10^5^ cells/well. To assess chemokine usage, PBMCs and GHOST cell lines were inoculated with equal amounts of each virus (100 TCID_50_ in a final volume of 100 μl/well) and incubated for 3 h/37°C in the presence of 3 μg/ml of Polybrene. Cells were then washed and cultured in appropriate culture medium (500 μl/well). Viral replication was monitored in culture supernatants by RT activity by an enzyme-linked immunosorbent assay (Lenti-RT kit, Cavidi) during 12-day period after infection. Additionally, in some experiments, viral infection in GHOST cells was also monitored by LTR-driven GFP expression as described [7].

### Susceptibility to CCR8 blockade

The chemokine I-309, specific for CCR8 [68,71], was purchased from R&D Systems (Minneapolis, MN). HIV-2_MIC97_ and HIV-2_MJC97_ sensitivity to I-309 was based on the inhibition of viral production as described [7,8]. Briefly, GHOST-CD4/CCR8 cells were seeded at 1.5 × 10^5^ cells per well in 24-well plates and allowed to adhere overnight. Cells were incubated for 1 h at 37°C with blocking concentrations (100 ng/ml) of I-309 [51]. Viruses were then added as described in infectivity assays and incubated for 4 h in an inhibitor-containing medium. Cells were washed with PBS to remove unadsorbed viral particles and cultured in an appropriate medium either containing the referred concentration of I-309. Alternatively, these inhibition assays were also performed using CD8-depleted PBMCs as target cells, in order to avoid any uncontrolled inhibition exerted by soluble factors eventually secreted by CD8+ T-cells. Virus production was assessed by RT activity in culture supernatants as described in infectivity assays. Viral production in the absence of inhibitor was used as control.

### *In vitro* adaptation experiments

The starting viruses for this study was obtained by transfection of 293 T cells with the pROD/MIC-SB and pROD/MJC-SB plasmids [52]. These plasmids contain an infectious HIV-2_ROD_ provirus into which the *env* gene derived from both HIV-2_MIC97_ and HIV-2_MJC97_ isolates, was cloned [52]. The cells used in this experiment were the GHOST-CD4 cell lines individually expressing CCR8, CCR5 or CXCR4. An initial stock of each virus (ROD/MIC-SB and ROD/MJC-SB) was prepared by passing the virus-containing supernatants from transfected 293 T cells in GHOST-CD4/CCR8 cells. Each virus was then used to infect a 90:10 (%) mixture of GHOST-CD4/CCR8:GHOST-CD4/CCR5 and GHOST-CD4/CCR8:GHOST-CD4/CXCR4 in the presence of 3 μg/ml of Polybrene. The infection of the 90:10 GHOST cells mixture was done by spinoculation in order to further enhance the efficiency of virus binding to target cell [114]. At day 12 after infection, culture supernatants were used to infect either a pure population of GHOST-CD4/CCR5 (or GHOST-CD4/CXCR4) cells, or an 80:20 mixture of GHOST-CD4/CCR8:GHOST-CD4/CCR5 and GHOST-CD4/CCR8:GHOST-CD4/CXCR4 in the same conditions referred for initial 90:10 cell mixtures. Virus-containing supernatant from these latter cultures was again used to infect pure GHOST-CD4/CCR5 (or GHOST-CD4/CXCR4) or a 70:30 mixture of GHOST-CD4/CCR8:GHOST-CD4/CCR5 and GHOST-CD4/CCR8:GHOST-CD4/CXCR4. This procedure was repeated using cell mixtures with increasing proportions of GHOST-CD4/CCR5 or GHOST-CD4/CXCR4 cells, until a ratio 10:90 of GHOST-CD4/CCR8:GHOST-CD4/CCR5 or GHOST-CD4/CCR8:GHOST-CD4/CXCR4 cells. At day 12 after infection, viral replication in each cell mixture was assessed by RT activity in culture supernatants.

### Multi-site directed mutagenesis in the V1/V2 region of HIV-2_MJC97_

Site-directed mutagenesis was used to alter specific amino acid residues within V1/V2 region of HIV-2_MJC97_ SU envelope glycoprotein. Sequential mutations were introduced into plasmid pROD/MJC-SA which contains a HIV-2_MJC97_*env* fragment spanning from C1 to C4 region, inserted into genetic backbone of an infectious molecular clone of HIV-2_ROD_ strain [52]. Sequential codon changes were made using a QuickChange II XL site-directed mutagenesis kit, (Stratagene) and mutagenic primers listed in Table 3, according to manufacturer’s protocol. The presence of the desired mutations was confirmed by sequencing the C1-C4 region of each mutant.

**Table 3:** **Primers used in site-directed mutagenesis of V1V2 region of HIV-2**_**MJC97**_ **surface envelope glycoprotein**

**Primers**	Mutated residues*	**Orientation**	Sequence (5′ to 3′)**	Location***
**V1V2 Mut1**	K98N, S99I	+	GAGTTGTAACAA**C**A**TA**AGTGAAA	282–304
**V1V2 Mut1-R**		-	TTTCACT**TA**T**G**TTGTTACAACTC	
**V1V2 Mut2**	N104T, S106T	+	AAACCACAA**CA**ACCA**CA**AGTAACAAC	302–327
**V1V2 Mut2-R**		-	GTTGTTACT**TG**TGGT**TG**TTGTGGTTT	
**V1V2 Mut3**	M147N	+	GTCAGTTCA**AC**ATGACAGGG	431–450
**V1V2 Mut3-R**		-	CCCTGTCAT**GT**TGAACTGAC	
**V1V2 Mut4**	Q160N	+	AAATCATAT**A**A**C**GAAACAT	469–487
**V1V2 Mut4-R**		-	ATGTTTC**G**T**T**ATATGATTT	
**V1V2 Mut5**	S114P, T116D, D117Q	+	ATCTATC**CC**CACA**GACC**A**G**TACAGC	333–357
**V1V2 Mut5-R**		-	GCTGTA**C**T**GGTC**TGTG**GG**GATAGAT	
**V1V2 Mut6**	Y118E, S119Q, L120E	+	GACCAG**G**A**GCAA**GAGATAAATGAGAGTTCTCC	346–377
**V1V2 Mut6-R**		-	GGAGAACTCTCATTTATCTC**TTGC**T**C**CTGGTC	
**V1V2 Mut7**	P172T, T173N, T176S	+	GTATGTGAA**A**CAA**AT**AATGAAA**GC**ACAAGCA	505–535
**V1V2 Mut7-R**		-	TGCTTGT**GC**TTTCATT**AT**TTG**T**TTCACATAC	
**V1V2 Mut5′**	I113P, S114G, T115S	+	ACAACATCT**CCAG**G**G**A**GC**ACA	328–351
**V1V2 Mut5′-R**		-	TGT**GC**T**C**C**CTGG**AGATGTTGT	
**V1V2 Mut6′**	D117L, Y118K, S119P	+	ACA**CTCA**A**ACC**CTTGATAAATGAGA	346–370
**V1V2 Mut6′-R**		-	TCTCATTTATCAAG**GGT**T**TGAG**TGT	
**V1V2 Mut7′**	T173F, E175T	+	TGTGAACCA**TTT**AAT**ACC**ACAACAAGC	508–534
**V1V2 Mut7′-R**		-	GCTTGTTGT**GGT**ATT**AAA**TGGTTCACA	

*and ***Numbers of amino acid residues or nucleotides are referred to HIV-2_MJC97_ sequence (GenBank accession number: EU021092). **Mutations in the primer sequence are represented in boldface.

Virus particles were produced by transfecting 293 T cells with purified DNA from each mutated constructs, using FuGENE6 transfection reagent (Roche) according to manufacturer’s instructions and as described [52]. Viral stocks of mutated viruses were prepared by passaging each viral-containing supernatants from transfected 293 T cells in IL2-stimulated PBMCs. The TCID_50_ of each viral stock was determined in PBMCs.

To assess replication competence and coreceptors usage of wild type or mutated viruses, PBMCs and GHOST cell lines were inoculated with titrated viral stocks according to the protocol described in “Infectivity assays” section.

### Statistical analysis

Statistical analysis was performed using Epi info version 6.04 (CDC, Atlanta, USA) and SPSS software version 10 (SPSS Inc, Chicago, USA). The univariate analysis was tested using χ2 and 2-tailed Fisher’s exact test in case of small sample size. Statistical significance was assumed when *p* < 0.05.

### Ethics statement

All healthy adult subjects (PBMC’s donors) provided written informed consent and validated by the Faculty of Pharmacy of Lisbon Institutional review board. None of the blood samples included in this study were gathered from infected patients.

## Availability of supporting data

The data sets supporting the results and methods of this article are available in the GenBank repository (http://www.ncbi.nlm.nih.gov/genbank); accession numbers: EU021092 (http://www.ncbi.nlm.nih.gov/nuccore/EU021092), AF082339 (http://www.ncbi.nlm.nih.gov/nuccore/AF082339) and M15390 (http://www.ncbi.nlm.nih.gov/nuccore/M15390).
